# Association of pre-pregnancy body mass index and gestational weight gain with neonatal anogenital distance in a Chinese birth cohort

**DOI:** 10.1186/s12978-022-01458-y

**Published:** 2022-06-29

**Authors:** Zhiyang Wang, Jinbo Niu, Honglei Ji, Maohua Miao, Limei Yang, Xia Chen, Xiufeng Li, Xiuxia Song, Aimin Chen, Hong Liang, Wei Yuan

**Affiliations:** 1 NHC Key Lab. of Reproduction Regulation, Shanghai Institute for Biomedical and Pharmaceutical Technologies, #779 Lao Hu Min Road, Shanghai, 200032 China; 2 Department of Biological Sciences, University of Cincinnati, Cincinnati, OH 45221 USA; 3 The First People’s Hospital of Jiashan, Jiaxing, Zhejiang China; 4 Obstetrics and Gynecology Department, Maternity and Child Health Care Hospital, Jiashan County, Jiaxing, Zhejiang China; 5 Maternal Health Care Department, Maternity and Child Health Care Hospital, Jiashan County, Jiaxing, Zhejiang China; 6 Department of Biostatistics, Epidemiology and Informatics, Perelman School of Medicine, University of Pennsylvania, Philadelphia, PA 19104 USA

**Keywords:** Anogenital distance, Pre-pregnancy BMI, Gestational weight gain, Newborn

## Abstract

**Background:**

This study aimed to investigate the associations of pre-pregnancy body mass index (BMI) and gestational weight gain (GWG) with anogenital distance (AGD) among newborns.

**Methods:**

The study included 556 mother-newborn pairs from the Jiashan birth cohort. AGD was measured as AGD_AP_ (from the center of the anus to the anterior base of the penis, where the penile tissue meets the pubic bone) and AGD_AS_ (from the center of the anus to the posterior base of the scrotum, where the skin changes from rugate to smooth) in males and AGD_AC_ (from the center of the anus to the clitoris) and AGD_AF_ (from the center of the anus to the posterior convergence of the fourchette) in females. Multiple linear regression models were used to estimate the associations of pre-pregnancy BMI and GWG, with AGD.

**Results:**

After adjusting for pre-pregnancy BMI and other potential confounders, male newborns whose mothers had excessive GWG had shorter AGD_AP_ than those whose mothers had normal GWG. Male newborns whose mothers had normal pre-pregnancy BMI and inadequate/excessive GWG had shorter AGD_AP_ than the reference group where mothers had normal pre-pregnancy BMI and GWG in stratified analyses.

**Conclusion:**

Gestational weight gain during pregnancy was associated with AGD in newborns in this birth cohort.

**Supplementary Information:**

The online version contains supplementary material available at 10.1186/s12978-022-01458-y.

## Plain language summary

In China, the prevalence of underweight and overweight/obesity remained high among women. Appropriate pre-pregnancy body mass index (BMI) and gestation weight gain (GWG) were critical to reduce the risk of adverse birth outcomes. The anogenital distance (AGD) was measured as an indicator of neonatal reproductive function and was associated with adverse reproductive outcomes in adults. Thus, we investigated the associations of both sub-optimal pre-pregnancy BMI, as well as GWG, with AGD among newborns to draw a picture about their effect on offspring reproductive health.

A total of 556 mother-newborns were included in the study from the Jiashan birth cohort in China. We extracted information about maternal lifestyles, social demographic characteristics, diet, and medical history from questionnaires conducted during 8–16 gestational weeks and medical records. AGD among newborns was measured within 3 days of delivery.

We found that maternal excessive GWG was associated with shorter AGD in male newborns after adjusting for maternal pre-pregnancy BMI in multiple linear regression models. The study also suggested that maternal inadequate GWG was associated with a shorter AGD in male newborns, which needed to be corroborated in further studies with a larger sample size.

In conclusion, health professionals shall implement sufficient intervention to prevent suboptimal GWG during prenatal checkups.

**Supplementary Information:**

The online version contains supplementary material available at 10.1186/s12978-022-01458-y.

## Introduction

Pre-pregnancy underweight and overweight/obesity, measured by body mass index (BMI), is frequently used as a health indicator of women prior to pregnancy, and gestational weight gain (GWG) is one of the few modifiable risk factors for poor obstetric outcomes [1, 2]. In China, the frequency of pre-pregnancy underweight ranges from 11.04 to 16.3%, while that of pre-pregnancy overweight ranges from 18.3 to 18.64% and of pre-pregnancy obesity from 6.27 to 6.8% [3–5]. Both suboptimal pre-pregnancy BMI and GWG are associated with a number of pregnancy complications and adverse birth outcomes. Pre-pregnancy overweight and obesity are associated with increased risks of gestational hypertension, gestational diabetes mellitus, and macrosomia [6, 7], while pre-pregnancy underweight and inadequate GWG are associated with higher risks of preterm birth and low birth weight (LBW) [8, 9].

The altered reproductive function was also found to be associated with pre-pregnancy BMI and GWG. Animal studies have suggested that maternal pre-pregnancy obesity is associated with lower testosterone and luteinizing hormone (LH) levels, increased testicular and sperm oxidative stress, increased sperm DNA fragmentation, and a higher level of aberrant sperm chromatin [10, 11]. Meanwhile, low body fat content in female mice can induce the stimulatory action of follicle stimulation hormone on ovarian progesterone to suppress reproduction [12]. Epidemiological studies have reported that the daughters of overweight mothers had an earlier age of menarche and lower levels of estradiol and free estrogen index (FEI) in adulthood [13].

As a sensitive indicator of intrauterine hormone disruption, the anogenital distance (AGD) is defined as the distance from the anus to the genital tubercle. In the Environmental Protection Agency testing guidelines, AGD was added as an endpoint for reproductive toxicity in 1996 [14]. In humans, shorter AGD has been associated with adverse reproductive outcomes, including poorer semen quality [15], lower female fertility [16], undescended testis [17], and hypospadias [18], suggesting that AGD could be a good indicator of neonatal and adult reproductive function [19]. A recent study indicated that pre-pregnancy BMI was positively correlated with AGD in male fetuses, as measured by ultrasound during pregnancy. However, ultrasound measurement only covered a range of gestational age between 26 and 30 weeks [20].

This study aimed to investigate the associations of both pre-pregnancy underweight and overweight or obesity, as well as inadequate and excessive GWG, with AGD among newborns in a birth cohort from the Jiashan County, China.

## Method

### Study participants and design

**Fig. 1:**
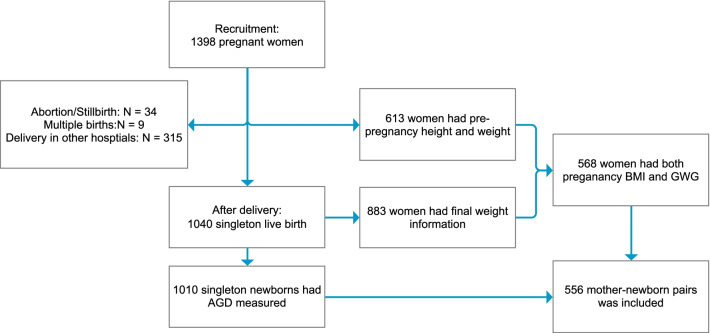
Study population of the present study from the Jiashan birth cohort

Pregnant women who visited the First People's Hospital of the Jiashan County and Women and Children`s Hospital in the Jiashan County for their first prenatal care at 8–16 weeks of gestation were enrolled in the Jiashan birth cohort from September 2016 to April 2018. Women who were native Chinese and residents of Jiashan County; had no hospital-diagnosed major chronic diseases, including hypertension, hyperlipidemia, gallbladder disease, thyroid disease, diabetes, and hepatitis; intended to complete scheduled interviews during pregnancy and after delivery; and planned to give birth at the First People's Hospital of Jiashan County were invited to participate in the study. A total of 1398 eligible pregnant women were recruited in the cohort. During the follow-up, 358 women left the cohort due to abortion/stillbirth (34), multiple births (9) and delivery in other hospitals (315). A total of 1010 live singletons were born in the First People's Hospital of Jiashan County and had AGD measurements (Fig. 1). Information on maternal lifestyles, social demographic characteristics, diet, and medical history was collected using structured questionnaires at recruitment. We extracted information on the neonate`s birth date, birth weight, sex, maternal gestational age, height, pre-pregnancy weight, and weight before delivery from medical records.

All mothers gave written informed consent at enrollment for themselves and their children. This study was approved by the ethical committees of the Shanghai Institute of Planned Parenthood Research (IRB00008297).

### Maternal BMI and GWG assessment

Pre-pregnancy BMI (kg/m^2^) was calculated as the ratio of the weight (kg) divided by height squared (m^2^). At recruitment, a total of 472 women provided information on pre-pregnancy weight and height. In addition, information on women’s weight before 8 weeks of gestation (if present) and height was extracted from medical records and the weight was used as a proxy for the pre-pregnancy weight (n = 141).

In total, there were 613 pregnant women who had information on pre-pregnancy BMI (Fig. 1). According to the criteria in the guidelines for prevention and control of overweight and obesity in Chinese adults, the pre-pregnancy BMI was categorized into three groups: underweight (BMI < 18.5 kg/m^2^), normal (18.5 kg/m^2^ ≤ BMI < 24.0 kg/m^2^), and overweight/obesity (BMI ≥ 24.0 kg/m^2^) [21]. Due to the low frequency of women with a BMI over 30 kg/m^2^ (2.94%), we did not define a separate group for obese women.

GWG was calculated as the difference between the measured weight in final prenatal care (between 37 and 41 gestational weeks), no more than two weeks before delivery, and pre-pregnancy weight. The final weight was extracted from the medical records. We excluded preterm delivery (n = 10) and post-term delivery (n = 2) from data analysis. A total of 568 eligible women had information of both pre-pregnancy BMI and GWG. In accordance with the guidelines of the Institute of Medicine, the GWG were categorized into three groups: inadequate GWG, normal GWG, and excessive GWG [22]. The recommended normal GWG varied according to pre-pregnancy BMI, ranging from 12.5 to 18.0 kg for pre-pregnancy underweight women, from 11.5 to 16.0 kg for pre-pregnancy normal weight women, and from 7 to 11.5 kg for pre-pregnancy overweight women.

Finally, a total of 556 mother-infant pairs who had information on both infant’s AGD and maternal pregnancy BMI and GWG, were included in this prospective study (Fig. 1).

### Measurements of AGD in newborns

In males, AGD_AP_ (from the center of the anus to the anterior base of the penis, where the penile tissue meets the pubic bone) and AGD_AS_ (from the center of the anus to the posterior base of the scrotum, where the skin changes from rugate to smooth) were measured. In females, AGD_AC_ (from the center of the anus to the clitoris) and AGD_AF_ (from the center of the anus to the posterior convergence of the fourchette) were measured (Additional file 1: Fig. S1) [23–25].

In the study, the physicians used the standardized caliper to measure AGDs of all newborns, and all AGD measurements were conducted within 3 days of delivery of newborns. In measurement, the newborns were positioned with the legs held back in a frog leg posture at a 60–90° angle from the torso at the hip by an assistant. The physicians stood in front of the newborn and made independent measurements of AGD using the same digital caliper [23].

In order to standardize the measurements, the physicians were trained to use the standardized caliper to conduct AGD measurements without knowledge of the participants` exposure status [26]. Two physicians completed AGD measurements of all newborns, while the AGD of each newborn was only measured once. However, two physicians independently performed AGD measurements of 30 same newborns. Based on the data, the intraclass correlation coefficients (ICC) of AGD_AP_, AGD_AS_, AGD_AC_, and AGD_AF_ were 0.836, 0.731, 0.624, and 0.722, respectively, which indicated moderate to good inter-rater reliability for AGD measurements [23]. We did not evaluate within-examiner variations, but previous studies suggested that measurement error due to within-examiner variation was low and further measurements could not gain more reliability [27, 28].

### Data analysis

Multiple linear regression models were used to examine the associations of pre-pregnancy BMI and GWG with AGD (used as continuous in all analyses). Potential confounding variables were identified a priori based on their relationship with AGD. We adjusted for maternal age at conception (≤ 25, 25–29, > 30 years), gestational weeks at birth, maternal education (primary school or below, middle school, high school, and college or higher), parity (0, ≥ 1), maternal folic acid intake during pregnancy (yes or no), passive smoking (yes or no), newborn birth weight, GWG (inadequate, normal, excessive, only for pre-pregnancy BMI analysis), and pre-pregnancy BMI (underweight, normal, overweight or obesity, only for GWG analysis) [25, 29]. Regression coefficients and 95% confidence intervals (95% CIs) were reported.

To examine the separate effects of BMI and GWG, we reported the estimates of BMI or GWG while holding the other in a fixed group (normal group) using multiple linear regression models. Subjects were further divided into five groups: normal pre-pregnancy BMI and inadequate GWG, normal pre-pregnancy BMI and excessive GWG, pre-pregnancy underweight and normal GWG, pre-pregnancy overweight and normal GWG, and normal pre-pregnancy BMI and normal GWG group. The last group was used as the reference group, and the first four groups were placed in the multiple linear regression model at the same time:

$${\text{AGDs}}\,{ = }\,\beta_{0} + \beta_{1} X_{1} + \beta_{2} X_{2} + \beta_{3} X_{3} + \beta_{4} X_{4} + \cdots$$

X_1_: normal pre-pregnancy BMI with inadequate GWGX_2_: normal pre-pregnancy BMI with excessive GWG.

X_3_: pre-pregnancy underweight with normal GWG X_4_: pre-pregnancy overweight or obesity with normal GWG.

The sample size of groups with both pre-pregnancy BMI and GWG outside of the normal range did not provide sufficient power to detect a difference; thus, we did not include the group in the analyses (Additional file 1: Table S1).

Because of the sexual difference in AGD, statistical analyses were stratified by newborn sex. All analyses were conducted using SAS 9.4 (SAS Institute Inc., Cary, NC, USA).

## Results

### Demographic characteristics

The demographic characteristics of the study subjects according to pre-pregnancy BMI are presented in Table 1. The normal BMI group (n = 356) accounted for 64.03% of the pregnant women studied. There were 94 pregnant women in the underweight group and 106 in the overweight or obesity group. The majority of the pregnant women took folic acid supplementation during pregnancy (88.48%) and were multiparous (68.17%) in this study. The percentage of pregnant women during pregnancy exposed to passive smoking was 51.2% (n = 232).

**Table 1:** Demographic characteristics of subjects according to pre-pregnancy BMI

	N^a^	Mean ± SD / N (%)		
Maternal and Offspring`s Characteristics		Underweight (n = 94)	Normal BMI (n = 356)	Overweight or Obesity (n = 106)
Categorical variable				
Maternal age (years)				
≤25	181	41 (44.57%)	115 (32.76%)	25 (23.81%)
25–29	173	35 (38.04%)	107 (30.48%)	31 (29.52%)
> 29	194	16 (17.39%)	129 (36.75%)	49 (46.67%)
Education				
Primary school or below	16	0	12 (3.44%)	4 (3.85%)
Middle high school	166	24 (25.81%)	105 (30.09%)	37 (35.58%)
High school	129	20 (21.51%)	85 (24.36%)	24 (23.08%)
College or above	235	49 (52.69%)	147 (42.12%)	39 (37.50%)
Passive smoking				
Yes	232	43 (53.13%)	150 (51.37%)	39 (46.99%)
No	221	35 (44.87%)	142 (48.63%)	44 (53.01%)
Parity				
Nulliparous	177	42 (44.68%)	111 (31.18%)	24 (22.64%)
Multiparous	379	52 (55.32%)	245 (68.82%)	82 (77.36%)
Folic acid intake				
Yes	453	76 (88.37%)	287 (88.04%)	90 (90.00%)
No	59	10 (11.63%)	39 (11.96%)	10 (10.00)
Continuous Variable				
Gestational week	556	38.93 (1.06)	38.97 (1.03)	38.94 (1.06)
Infant weight (g)	511	3225.88 (334.76)	3360.02 (397.91)	3518.30 (394.89)

^a^There were 8, 10, 103, 44 and 45 missing values in maternal age, education, passive smoking, folic acid intake and infant birth weight, respectively

### Association between pre-pregnancy BMI and AGD in newborns

In the unadjusted analysis, compared with the normal pre-pregnancy BMI group, the pre-pregnancy overweight or obesity group had longer AGD_AP_ in male newborns (*β* = 1.68 mm, 95%CI: 0.16, 3.19), as shown in Table 2. In the adjusted analysis, we did not observe any significant associations between pre-pregnancy underweight or overweight/obesity and AGD indices. In addition, when pre-pregnancy BMI was used as a continuous variable in the model, there was still no significant association between pre-pregnancy BMI and AGD indices (Additional file 1: Table S3).

**Table 2:** Association between pre-pregnancy BMI and anogenital distance (AGD) in newborns

Anogenital Distance (mm)				
BMI Group	N^a^	Mean (SD)	Unadjusted β (95%CI)	Adjusted β (95%CI)^b, c^
AGD_AP_ in boys				
Underweight	56	47.58 (5.69)	0.80 (– 0.74, 2.34)	0.53 (– 1.30, 2.37)
Normal	202	46.78 (4.99)	Ref.	Ref.
Overweight or obesity	59	48.46 (5.36)	1.68 (0.16, 3.19)*	1.42 (-0.40, 3.25)
AGD_AS_ in boys				
Underweight	56	21.25 (4.85)	0.92 (– 0.42, 2.25)	1.55 (– 0.21, 3.30)
Normal	202	20.33 (4.25)	Ref.	Ref.
Overweight or obesity	59	20.83 (4.89)	0.50 (– 0.80, 1.81)	0.13 (– 1.61, 1.88)
AGD_AC_ in girls				
Underweight	48	35.44 (6.29)	0.94 (– 0.92, 2.80)	1.74 (– 0.47, 3.95)
Normal	178	34.50 (5.72)	Ref.	Ref.
Overweight or obesity	54	35.36 (5.69)	0.86 (-0.92, 2.64)	1.49 (– 0.58, 3.57)
AGD_AF_ in girls				
Underweight	48	13.99 (4.21)	0.71 (– 0.55, 1.97)	-0.32 (-1.88, 1.25)
Normal	178	13.28 (3.87)	ref	ref
Overweight or obesity	54	13.58 (3.89)	0.30 (– 0.90, 1.51)	0.03 (– 1.44, 1.51)

^a^The larger sample size in the unadjusted analysis was due to less missing information by only pre-pregnancy BMI and outcomes

^b^104 boys and 80 girls were not included in adjusted analyses due to missing values in covariates

^c^Adjusted for maternal gestational weight gain, maternal age at conception, gestational weeks at birth, education, parity, folic acid intake during pregnancy, passive smoking and infant birth weight

*p < 0.05

### Association between GWG and AGD in newborns

In the unadjusted analysis, compared with the normal GWG group, no significant association between inadequate or excessive GWG and AGD was found, as shown in Table 3.

**Table 3:** Association between maternal gestational weight gain and anogenital distance (AGD) in newborns

Anogenital Distance (mm)				
GWG group	N	Mean (SD)	Unadjusted β (95%CI)	Adjusted β (95%CI)^a, b^
AGD_AP_ in boys				
Inadequate	80	46.34 (5.40)	− 1.21 (− 2.65, 0.22)	− 1.56 (− 3.25, 0.13)
Normal	126	47.55 (4.23)	Ref.	Ref.
Excessive	92	47.30 (5.88)	− 0.25 (− 1.63, 1.13)	− 2.27 (− 3.92, − 0.62)*
AGD_AS_ in boys				
Inadequate	80	19.97 (5.18)	− 0.92 (− 2.19, 0.36)	− 1.00 (− 2.62, 0.61)
Normal	126	20.88 (4.13)	Ref.	Ref.
Excessive	92	20.84 (4.50)	− 0.05 (− 1.27, 1.18)	− 0.67 (− 2.25, 0.91)
AGD_AC_ in girls				
Inadequate	77	33.63 (4.92)	− 1.29 (− 2.96, 0.37)	− 0.79 (− 2.67, 1.10)
Normal	108	34.92 (5.73)	Ref.	Ref.
Excessive	73	35.89 (6.26)	0.97 (− 0.72, 2.66)	− 0.07 (− 1.99, 1.86)
AGD_AF_ in girls				
Inadequate	77	13.50 (3.96)	0.06 (− 1.09, 1.21)	− 0.62 (− 1.96, 0.72)
Normal	108	13.44 (3.74)	Ref.	Ref.
Excessive	73	13.38 (4.14)	− 0.06 (− 1.23, 1.11)	− 0.76 (− 1.96, 0.72)

^a^85 boys and 58 girls were not included in adjusted analyses due to missing values in covariates

^b^Adjusted for maternal pre-pregnancy BMI, maternal age at conception, gestational weeks at birth, education, parity, folic acid intake during pregnancy, passive smoking and infant birth weight

*p < 0.05

The adjusted regression model showed AGD_AP_ in male newborns decreased by 2.27 mm in the excessive GWG group (95%CI: -3.92, -0.62), compared with the normal GWG group. We did not observe any significant association in female newborns.

### The separate effects of pre-pregnancy BMI and GWG on AGD in newborns in stratified analyses

As shown in Table 4, after adjusting for potential confounders, male newborns in the normal pre-pregnancy BMI and excessive GWG group had shorter AGD_AP_ (β = -2.65 mm, 95%CI: -4.66 -0.64), compared with the reference group of normal pre-pregnancy weight and normal GWG. Furthermore, we found that male newborns in the normal pre-pregnancy BMI and inadequate GWG group had shorter AGD_AP_ (β = -2.64 mm, 95%CI: -4.59, -0.69).

**Table 4:** Association between pre-pregnancy BMI or GWG and anogenital distance (AGD) in newborns in stratified analyses

		Anogenital Distance (mm)	
Joint Effect Group	N	unadjusted β (95%CI)	adjusted β (95%CI)^a, b^
AGD_AP_ in boys			
Normal pre-pregnancy BMI with normal GWG	86	Ref.	Ref.
Normal pre-pregnancy BMI with inadequate GWG	56	− 2.26 (− 3.84, − 0.67)*	− 2.64 (− 4.59, − 0.69)*
Normal pre-pregnancy BMI with excessive GWG	48	− 1.05 (− 2.71, 0.61)	− 2.65 (− 4.66, − 0.64)*
Pre-pregnancy underweight with normal GWG	26	− 1.37 (− 3.44, 0.69)	− 1.39 (− 3.88, 1.11)
Pre-pregnancy overweight or obesity with normal GWG	14	1.40 (− 1.26, 4.06)	0.90 (− 2.66, 4.46)
AGD_AS_ in boys			
Normal pre-pregnancy BMI with normal GWG	86	Ref.	Ref.
Normal pre-pregnancy BMI with inadequate GWG4	56	− 1.71 (− 3.18, − 0.24)*	− 1.58 (− 3.46, 0.30)
Normal pre-pregnancy BMI with excessive GWG 6	48	0.33 (− 1.22, 1.87)	− 0.17 (− 2.10, 1.77)
Pre-pregnancy underweight with normal GWG 2	26	0.37 (− 1.54, 2.29)	1.47 (− 0.93, 3.87)
Pre-pregnancy overweight or obesity with normal GWG 8	14	0.08 (− 2.39, 2.55)	− 0.42 (− 3.85, 3.01)
AGD_AC_ in girls			
Normal pre-pregnancy BMI with normal GWG	75	Ref.	Ref.
Normal pre-pregnancy BMI with inadequate GWG	45	− 0.19 (− 2.30, 1.92)	− 0.17 (− 2.60, 2.26)
Normal pre-pregnancy BMI with excessive GWG	46	0.99 (− 1.11, 3.09)	− 0.32 (− 2.71, 2.07)
Prepregnancy underweight with normal GWG	20	2.79 (− 0.03, 5.61)	3.11 (− 0.29, 6.51)
Pre-pregnancy overweight or obesity with normal GWG	13	0.86 (− 2.51, 4.23)	0.23 (− 3.64, 4.11)
AGD_AF_ in girls			
Normal pre-pregnancy BMI with normal GWG	75	Ref.	Ref.
Normal pre-pregnancy BMI with inadequate GWG 4	45	0.17 (− 1.25, 1.59)	− 0.85 (− 2.53, 0.83)
Normal pre-pregnancy BMI with excessive GWG 6	46	− 0.26 (− 1.67, 1.15)	− 1.51 (− 3.16, 0.13)
Pre− pregnancy underweight with normal GWG 2	20	0.94 (− 0.96, 2.83)	− 1.03 (− 3.38, 1.31)
Pre-pregnancy overweight or obesity with normal GWG 8	13	− 0.52 (− 2.79, 1.74)	− 1.26 (− 3.94, 1.41)

^a^68 boys and 44 girls were not included in adjusted analyses due to missing values in covariates

^b^Adjusted for maternal age at conception, gestational weeks at birth, education, parity, folic acid intake during pregnancy, passive smoking and infant birth weight

*p < 0.05

## Discussion

This study provided preliminary epidemiological evidence that maternal excessive GWG was associated with shorter AGD in male newborns in both adjusted and stratified analyses. We also found that shorter AGDs in male newborns associated with inadequate GWG only in stratified analyses. These results suggest that suboptimal GWG during pregnancy might adversely affect AGD in male newborns. Maternal pre-pregnancy weight and weight gain during pregnancy should be mindfully tracked and monitored through prenatal checks. Appropriate intervention could be given to women who have trends to have suboptimal weight gain during pregnancy.

In our study, no significant association was found between pre-pregnancy BMI and AGD in newborns. Only one previous study has observed a positive association between pre-pregnancy BMI and male fetal AGD, measured between 26 and 30 gestational weeks using ultrasound [20]. The fetuses were still in development and the varied subjects between the two studies may partly explain the different results. In addition, no associations between BMI and newborns AGD in the present study may be due to the small sample size of obese women before pregnancy, which limited the statistical power of the study.

To our knowledge, no study has ever reported the association between GWG and AGD. We observed that maternal excessive GWG was associated with shorter AGD in male newborns. AGD is an indicator of male reproductive tract masculinization and an endpoint for hormonally regulated sex differentiation [30, 31]. Intrauterine programming is affected by reproductive hormone levels [32], and AGD could be a marker of hormone disruption [33]. Maternal excessive GWG were associated with decreased maternal thyroid-stimulating hormone (TSH) and free thyroxine (FT4) levels [34], while decreased TSH and FT4 levels in cord blood serum were found to be associated with shorter AGD in male newborns [35]. Thyroid Hormones may affect sex hormone metabolism and synthesis, including but not limited to testosterone metabolism, secretion of gonadotropin-releasing hormone (GnRH), and the responses of LH and follicle-stimulating hormone (FSH) to GnRH administration, thus influencing the androgen function with ultimate consequences for AGD [35, 36]. Connecting these studies, we may suggest that excessive GWG has an impact on reducing AGD through the plausible mechanism via hormone changes. The mechanisms underlying the associations of GWG with AGD in offspring still remain unclear and elusive, and further studies are needed to explore the potential mechanisms.

We also found a shorter AGD in male newborns whose mothers had inadequate GWG. However, the finding was only observed in stratified analyses with a small sample size, which had a limited power. Larger studies are warranted to corroborate the findings of this study.

Our study includes several strengths. First, AGD in both males and females shortly after birth was measured, each with two AGD indicators. Second, we evaluated the effects of both pre-pregnancy underweight and overweight or obesity, as well as inadequate and excessive GWG, which is particularly meaningful under the circumstance of a large percentage of underweight women in China and East Asia countries [5, 37].

This study has several limitations. Many participants (44.95%) in the cohort were not included in the analysis due to missing information on pre-pregnant BMI and GWG, which may produce selection bias. However, the excluded mother-infant pairs had similar sociodemographic characteristics including maternal age, education, and passive smoking to those excluded (Additional file 1: Table S2), which reduced our concern about selection bias. Another limitation is about active smoking. We did not include active smoking as a confounding variable because the proportion of active female smokers in China is small, from 2.6% to 2.7% [38, 39], and much less in pregnant women. Additionally, the potential measurement error in AGD was more likely to cause nondifferential misclassification, and it would bias the association toward null. Finally, the number of women in each group in the stratified effect analyses was small (Additional file 1: Table S1). Thus, stratified group analysis may only provide a rudimentary picture for the separate effects of pre-pregnancy BMI and GWG on AGD.

## Conclusion

Our findings provide preliminary evidence that maternal excessive GWG was associated with lower AGD in male newborns. These associations could draw a picture that suboptimal GWG might adversely affect with offspring reproductive health. Further studies are needed to validate these results. Our findings strengthen the notion that appropriate interventions should be taken to maintain normal GWG during pregnancy. Health professionals might be aware of our findings and guide women to maintain proper weight during counseling for couples intending to conceive. Timely interventions should also be taken to prevent both inadequate and excessive GWG during prenatal checkups.

## Supplementary Information

**Additional file 1: Table S1.** Number of subjects in separated groups in adjusted joint analyses. **Table S2.** Demographic characteristics of the included and excluded mother-newborn pairs. **Table S3.** Association between continuous pre-pregnancy BMI and anogenital distance (AGD) in newborns. **Figure S1.** Diagram of anatomical anogenital distance measurements in both boys and girls (adapted from [25]).

## Data Availability

The de-identified data are available upon reasonable request to the corresponding author.

## Abbreviations

BMI: Body mass index
GWG: Gestational weight gain
AGD: Anogenital distance
LBW: Low birth weight
LH: Luteinizing hormone
FEI: Free estrogen index
ICC: Intraclass correlation coefficients
TSH: Thyroid stimulating hormone
FT4: Free thyroxine
GnRH: Gonadotropin-releasing hormone
FSH: Follicle-stimulating hormone
