# Supplementary material for: Association of pre-pregnancy body mass index and gestational weight gain with neonatal anogenital distance in a Chinese birth cohort
Source: Reprod Health. 2022 Jun 29;19:152. doi: 10.1186/s12978-022-01458-y (PMC9245211; doi:10.1186/s12978-022-01458-y)

**Contents of Additional file 1**

Table S1: Number of subjects in separated groups in adjusted joint analyses

Table S2: Demographic characteristics of the included and excluded mother-newborn pairs

Table S3: Association between continuous pre-pregnancy BMI and anogenital distance (AGD) in newborns

Figure S1: Diagram of anatomical anogenital distance measurements in both boys and girls (adapted from (25))

Table S1: Number of subjects in separated groups in adjusted joint analyses

| **N (%)** | | | | |
| --- | --- | --- | --- | --- |
| **Boys** | | | | |
| N=213 | Pre-pregnancy BMI | | | |
|  |  | Underweight | normal | Overweight or Obesity |
| Gestational Weight Gain | Inadequate | **9 (4.23%)** | 38 (17.84%) | **8 (3.76%)** |
|  | normal | 19 (8.92%) | 60 (28.17%) | 8 (3.76%) |
|  | Excessive | **10 (4.69)** | 37 (17.37%) | **24 (11.27%)** |
| **Girls** | | | | |
| N=200 | Pre-pregnancy BMI | | | |
|  |  | Underweight | normal | Overweight or Obesity |
| Gestational Weight Gain | Inadequate | **13 (6.5%)** | 33 (16.50%) | **11 (5.50%)** |
|  | normal | 13 (6.5%) | 60 (30.00%) | 11 (5.50%) |
|  | Excessive | **4 (2.00%)** | 38 (19.00%) | **17 (8.50%)** |

Table S2: Demographic characteristics of the included and excluded mother-newborn pairs

| **Characteristics** | **Included mother-newborn pairs N (%)** | **Excluded mother-newborn pairs**  **N (%)** |
| --- | --- | --- |
| **Maternal age (years)** | | |
| <=25 | 181 (33.03%) | 118 (28.23%) |
| 25-29 | 173 (31.57%) | 152 (36.36%) |
| > 29 | 194 (35.40%) | 148 (35.41%) |
| **Education** |  |  |
| Primary school or below | 16 (2.93%) | 9 (2.08%) |
| Middle high school | 166 (30.40%) | 120 (27.71%) |
| High school | 129 (23.63%) | 88 (20.32%) |
| College or above | 235 (43.04%) | 216 (49.88%) |
| **Passive smoking** | | |
| Yes | 232 (51.21%) | 80 (40.20%) |
| No | 221 (48.79%) | 119 (59.80%) |
| **Parity** |  |  |
| Nulliparous | 177 (31.83%) | 129 (28.99%) |
| Multiparous | 379 (68.17%) | 316 (71.01%) |
| **Folic acid intake** | | |
| Yes | 453 (88.48%) | 372 (89.86%) |
| No | 59 (11.52%) | 42 (10.14%) |

Table S3: Association between continuous pre-pregnancy BMI and anogenital distance (AGD) in newborns

| **Anogenital Distance (mm)** | | | |
| --- | --- | --- | --- |
| **BMI** | **N**^a^ | **unadjusted β (95%CI)** | **adjusted β (95%CI)^b, c^** |
| AGD_AP_ in Male | 317 | 0.10 (-0.09, 0.29) | 0.08 (-0.15, 0.31) |
| AGD_AS_ in Male | 317 | -0.01 (-0.18, 0.15) | -0.15 (-0.37, 0.07) |
| AGD_AC_ in Female | 280 | 0.01 (-0.18, 0.19) | 0.09 (-0.15, 0.34) |
| AGD_AF_ in Female | 280 | 0.02 (-0.10, 0.15) | 0.14 (-0.03, 0.31) |

^a^ The larger sample size in the unadjusted analysis was due to less missing information by only pre-pregnancy BMI and outcomes.

^b^ 104 boys and 80 girls were not included in adjusted analyses due to missing value in covariates.

^c^ Adjusted for maternal gestational weight gain, maternal age at conception, gestational weeks at birth, education, parity, folic acid intake during pregnancy, passive smoking and infant birth weight.

Figure S1: Diagram of anatomical anogenital distance measurements in both boys and girls (adapted from (25)).


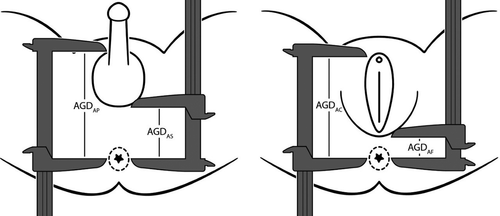

Supplement: Supplementary file 1 — Additional file 1: Table S1. Number of subjects in separated groups in adjusted joint analyses. Table S2. Demographic characteristics of the included and excluded mother-newborn pairs. Table S3. Association between continuous pre-pregnancy BMI and anogenital distance (AGD) in newborns. Figure S1. Diagram of anatomical anogenital distance measurements in both boys and girls (adapted from [25]). [file 12978_2022_1458_MOESM1_ESM.docx]
